# A Well-Merited Recognition

**Published:** 1901-04

**Authors:** 


### A WELL-MERITED RECOGNITION.

We cordially indorse the reappointment, just announced, of State Veterinarian Knowles, of Montana. We sincerely thank the appointing power for this acknowledgment of work well done for that State, and congratulate the people, the live-stock owners, and the agriculturists of that commonwealth on the retention of so capable and efficient a public servant. This good work speaks for itself in the excellent police sanitary regulations now prevailing in that State, the restriction of losses from preventable communicable animal diseases, and now one of the most comprehensive State laws regulating meat-inspection and milk-inspection ever adopted. Our continued good wishes to our esteemed colleague Knowles.

---

On January 29, 1901, Christopher Hope died at Carnegie, Pa. Dr. Hope was a registered practitioner, and had attained the age of seventy-five years.

